# Barriers and Facilitators in Implementing Clinical Practice Guidelines Among Nurses in Emergency Departments and Critical Care Units: A Systematic Review

**DOI:** 10.1111/jocn.70086

**Published:** 2025-08-24

**Authors:** Mona Hamdi Alrashdi, Barry Quinn, Susan A. Clarke

**Affiliations:** ^1^ Queen's University Belfast Belfast UK; ^2^ Taibah University Medina Saudi Arabia

**Keywords:** barriers, clinical guidelines, critical care, emergency department, facilitators, nursing, systematic review

## Abstract

**Aim:**

This systematic review explored the barriers and facilitators to the implementation of clinical practice guidelines (CPGs) among nurses in emergency departments (EDs) and critical care units (CCUs).

**Design:**

Systematic review.

**Method:**

A systematic review was performed using both qualitative and quantitative studies from five databases (CINAHL, Web of Science, Embase, Scopus and PubMed). The literature search was conducted in May 2024. The PRISMA framework was used to guide the review process. Findings were subject to a narrative, thematic analysis and critical appraisal.

**Results:**

Eighteen studies were identified that met the inclusion criteria, yielding three themes related to barriers and facilitators of guideline implementation: individual‐level, guideline‐level and organisational‐level. Key barriers included lack of awareness of guidelines, lack of knowledge and skills, attitudes towards guidelines, resource limitations, lack of perceived support, complexity of guidelines and lack of training. Facilitators to guideline use included colleague support, adequate training, effective leadership and refinement of guidelines to ensure relevance, local adaptation and user‐friendly content.

**Discussion:**

Numerous barriers to nurse implementation of CPGs exist in ED and CCU settings, reflecting a complex interplay of individual, CPG‐related and organisational factors. To facilitate CPG implementation, it is important for staff to be educated and trained in their use, supported to implement (including resource allocation) and that CPGs are designed to be easily implemented in practice.

**Conclusion:**

This systematic review highlights risk factors for poor CPG implementation and highlights the importance of addressing awareness, knowledge, resources and support for CPG use through targeted training, leadership and CPG design.

**Relevance to Clinical Practice:**

An analysis of barriers and facilitators to CPG implementation among nurses in ED and CCU settings provides an important opportunity to address a gap in the literature, facilitating the development of strategies to promote CPG use and enhance care quality among nurses in these specific contexts.

**Summary**

- This systematic review explores barriers and facilitators influencing clinical practice guideline (CPG) implementation among nurses in emergency and critical care settings.
- Barriers are shaped by three themes: individual factors (awareness, education, skills), CPG‐related factors (clarity, complexity, usability), and organisational factors (resources, workload, leadership support).
- Facilitators include targeted education, structured training, supportive leadership, clear roles, and adequate resource allocation.
- Findings emphasise the need for simplified, well‐designed guidelines that are adaptable to practice environments.
‐Enhancing adherence to CPGs can improve standardisation of care and patient outcomes in emergency and critical care units.

## 1 Introduction

Quality and safety in healthcare are a key priority across health settings, and a range of tools may be used to promote quality and consistency in practice. Examples of quality tools include care bundles, checklists and clinical practice guidelines (CPGs). The use of CPGs is a crucial element of evidence‐based care delivery, providing recommendations to inform decision‐making and the potential application of other tools, such as checklists and care bundles. By promoting adherence to best practices, CPGs help standardise care delivery, reduce variability and improve both patient safety and treatment outcomes (De Leo et al. 2023). As CPGs are developed by expert panels, utilising the best available evidence and considering resource needs to streamline care delivery, their implementation is considered imperative to deliver high‐quality nursing care in modern practice (World Health Organisation 2025). Indeed, adherence to CPGs is associated with improved patient care and safety, as well as reductions in patient morbidity, mortality, care costs and inconsistency in practice (Moore et al. 2017; Wager et al. 2021). By standardising evidence‐based interventions, CPGs enhance the overall clinical efficiency, minimising the possibility of errors and contributing to better patient outcomes (Moore et al. 2017; Wager et al. 2021).

Despite the recognised importance of CPGs in promoting best practice in care, the implementation of these guidelines is prone to variability (Kirk et al. 2016; Reich et al. 2018; Teggart et al. 2022). Low adoption of CPGs may lead to patients not receiving high‐quality care and increases in morbidity and mortality (Tudor Car et al. 2019; White et al. 2019). The adoption of CPGs may be subject to significant heterogeneity in different contexts, highlighting the importance of evaluating specific care settings when evaluating the potential for barriers and facilitators to guideline uptake (Moore et al. 2017; Tudor Car et al. 2019).

In the context of acute care, the use of CPGs may be particularly important in standardising approaches to rapid decision‐making in settings where patients have life‐threatening illnesses, including the emergency department (ED) (Kuhn et al. 2015). Furthermore, care of patients who are acutely unwell also occurs in critical care units (CCUs), inclusive of high dependence units (HDUs) and intensive care units (ICUs), where ongoing care of critically unwell patients may be facilitated (Kuhn et al. 2015). Within these settings, specific barriers may be present that impair the adoption of CPGs; for example, a lack of time due to high levels of workload, a busy ward environment and stress‐related factors that place pressure on staff to make decisions rapidly (Sole et al. 2020). In particular, nursing staff in these settings may be prone to high workloads and stress, which is problematic as nurses tend to have the highest level of patient contact of all staff and play key roles in care delivery and decision‐making (International Council of Nurses 2020). Hence, the importance of nurses adhering to CPG recommendations in these settings cannot be overstated; however, there may be specific challenges to the adoption and implementation of guidelines in this group (Sole et al. 2020).

While studies have sought to explore barriers and facilitators to CPG implementation, many of these focus on heterogeneous populations and groups rather than focusing on staff within specific care settings, or nursing staff in particular. There is a clear need to evaluate barriers and facilitators to CPG implementation by nurses in ED and CCU contexts to overcome this gap in the knowledge.

## 2 Aim of the Study

The aim of this systematic review was to identify known barriers and facilitators to the implementation of CPGs among nurses in ED and CCU settings, including ICU and HDU settings.

## 3 Methods

A systematic literature review (SLR) methodology was used in this study in order to provide a structured, transparent and reliable integration and synthesis of the scientific literature on this topic (Booth et al. 2021). The SLR approach was favoured due to the potential to minimise bias compared with other review types, while also providing the most robust and inclusive analysis of data on this topic, which may have particular value for practice (Booth et al. 2021). One key review question was derived to guide the review: ‘What are the barriers and facilitators to the implementation of CPGs among nurses in ED and CCU settings?’ The review question was influential in determining the search strategy and focus of the review. This type of review is empirical in nature, with the potential to combine qualitative and quantitative data from a multitude of research methodologies, thus forming a mixed methods SLR (Booth et al. 2021).

### 3.1 Databases

Five databases were used in this SLR: the Cumulative Index to Nursing and Allied Health Literature (CINAHL), Embase, PubMed, Scopus and Web of Science. Data from the Cochrane Library were also sought, but no systematic review data on the specific population or topic of interest was identified. The systematic search was conducted in May 2024.

Key search terms were combined with medical subject headings and Boolean operators to perform the search across all included databases (Table 1). Key search terms were derived from the ‘keywords’ of literature, wider reading to identify relevant synonyms relating to the population, exposure and outcomes of interest, as well as being developed in consultation with a subject expert librarian. In addition to the database search, a search was conducted of the reference lists/bibliographies of relevant papers.

**TABLE 1:** Search strategies used in electronic databases.

Database	Search term	Recorded
CINAHL	(“barriers” OR “obstacles” OR “facilitators” OR “enablers”) AND (“implementation”) AND (“clinical practice guidelines” OR “CPGs”) AND (“nurses”)	518
Embase	(barriers OR obstacles OR facilitators OR enablers) AND (implementation) AND (clinical practice guidelines OR clinical guidelines OR clinical guidelines OR CPGs) AND (nurses)	507
PubMed	(barriers OR obstacles OR facilitators OR enablers) AND (implementation) AND (clinical practice guidelines OR clinical guidelines OR clinical guidelines OR CPGs) AND (nurses)	1484
Scopus	(“barriers” OR “obstacles” OR “facilitators” OR “enablers”) AND (“implementation”) AND (“clinical practice guidelines” OR “CPGs”) AND (“nurses”)	797
Web of Science	(barriers OR obstacles OR facilitators OR enablers) AND (implementation) AND (clinical practice guidelines OR clinical guidelines OR clinical guidelines OR CPGs) AND (nurses)	1717

The inclusion criteria were developed based on the PCC (Population, Concept, Context) framework, focusing on registered nurses (Population), exploring barriers and facilitators to the implementation of clinical practice guidelines (Concept) within emergency and critical care clinical settings worldwide (Context).

Inclusion criteria for the review included the need for studies with nurses as participants or studies comprising a range of healthcare professionals, where nursing staff formed at least 50% of the total sample. Studies had to focus on facilitators or barriers to CPG implementation and should be performed exclusively in ED or CCU settings, including specialised settings such as paediatric, neonatal, or cardiac intensive care units. Studies had to be primary qualitative or quantitative studies (or mixed methods) and published in full in the English language. Studies were not eligible for inclusion if they focused on tools such as care bundles or local recommendations, rather than CPGs. No limits were placed on the year of study publication.

The preferred reporting items for systematic reviews or meta‐analyses (PRISMA), which is part of the EQUATOR Network, were used to guide the structure of the SLR (Moher et al. 2009). Following retrieval of the initial search results across the databases, references were imported into EndNote X9 software to facilitate management and refinement. A three‐step process was then undertaken to identify relevant studies for inclusion in the final data set. Firstly, duplicates were removed; secondly, the titles and abstracts of the remaining studies were screened using the Rayyan platform (http://rayyan.qcri.org), and finally, full‐text versions of the remaining studies were screened to confirm adherence to the inclusion criteria. Two reviewers (MA and SC) independently screened the studies to minimise the risk of bias and improve accuracy in study selection (Stoll et al. 2019). Any discrepancies between the two reviewers were resolved through discussion to achieve a consensus on the final data set.

The final data set (*n* = 18) underwent a formal process of data extraction using a structured data extraction tool/table. Parameters used to guide extraction included the study authors, date, objectives, design, setting, participants and key results (barriers and facilitators to implementation of CPGs). These parameters were agreed by the review team, reducing the potential for bias and error (Brown et al. 2006; Morton et al. 2011).

### 3.2 Quality Assessment

Quality assessment was completed for each of the studies using the Joanna Briggs Institute (JBI) checklist tools (Joanna Briggs Institute 2017). Three JBI checklists were used, comprising those for cross‐sectional studies (eight questions), randomised controlled trials (13 questions) and qualitative studies (10 questions). Individual scores were calculated by two independent reviewers (MA and BQ) and any sources of differences in scoring were discussed to resolve the issue and reach consensus on the final score. The final quality scores were based on positive responses to the checklist prompts/questions, as well as ‘not applicable’ criteria (Schultz et al. 2016).

A narrative approach was used to synthesise the findings of the studies in the final data set. This approach was favoured due to the heterogeneity of the data, which precluded meta‐analysis of purely quantitative integration strategies. A seven‐step approach was employed (Cooper 2019): problem formulation, literature search, gathering information, quality appraisal, analysis and integration of outcomes, evidence interpretation and presentation of the results. Specifically, the latter phases were facilitated by reading the studies in detail and comparing/contrasting the main findings (barriers and facilitators). Themes emerged from the literature based on the key trends and patterns that emerged within the data on comparison, with the review team collaborating to determine the final themes, generating reliable outcomes of the review process (Cooper 2019).

## 4 Results

**FIGURE 1:**
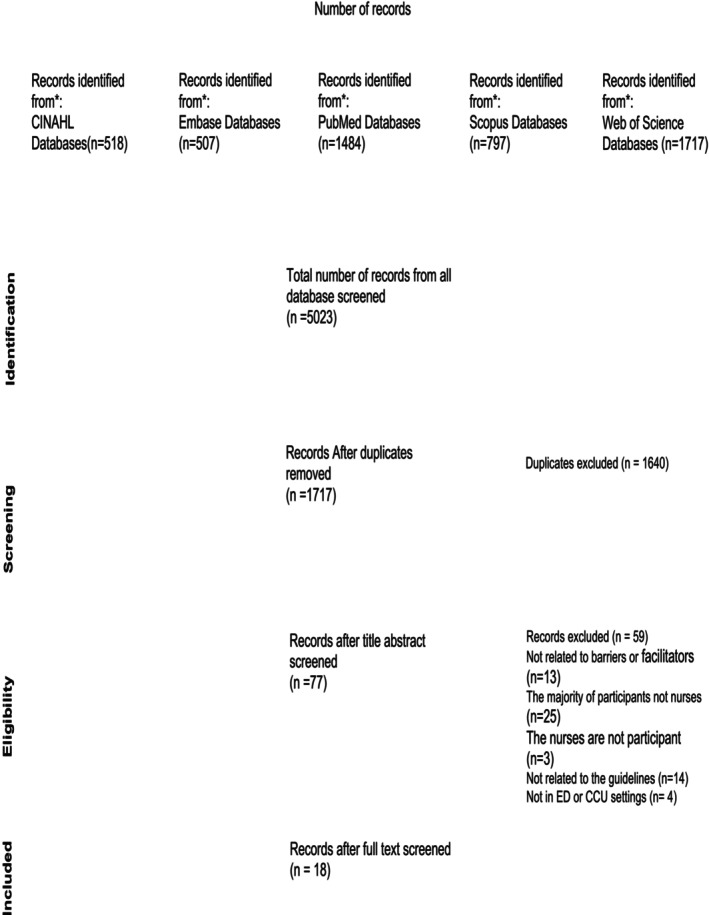
PRISMA flow diagram.

A summary of the search results and subsequent refinement phases is noted in Figure 1. The initial search results in 5023 studies across all databases, with 1717 unique studies identified after all duplicates were removed. After screening both the title and the abstract of these articles, 77 remained. The studies that were excluded at this stage were either not linked to the topic or did not focus on barriers and facilitators in the ED and CCU settings. A full‐text screening of the remaining studies led to the exclusion of 59 studies for the following reasons: the majority of participants were not nurses (*n* = 25), the study population did not include nurses (*n* = 3), were not related to guidelines (*n* = 14), assessed the implementation of CPGs without identifying barriers and/or facilitators (*n* = 13) or did not focus exclusively on ED or CCU settings (*n* = 4). The following screening and eligibility assessment allowed the identification of 18 studies that met the inclusion criteria, comprising 15 quantitative studies (14 cross sectional studies and one randomised controlled trial) and 3 qualitative studies (Figure 1. PRISMA diagram). The included studies covered research findings from multiple countries, including both high‐income (United States, Finland) and Tanzania as an example of a low‐income country. As a result, a diverse representation of different healthcare systems was ensured.

Of the indicated above 18 studies, 13 were focused on critical care units (CCUs), including high‐dependency units (HDUs) and intensive care units or ICUs; 4 were from emergency department settings (EDs), while the remaining study included nurses from both emergency and critical care settings. A summary of the characteristics of the included studies is presented in Table 2. The majority of studies were deemed ‘high quality’ (*n* = 15) while the remaining studies (*n* = 3) were classed as ‘moderate quality’ (Table 3).

**TABLE 2:** Summary of the included studies (*n* = 18).

Author, year and country	Aim	Design and method	Setting and sample	Barriers and facilitators
Al‐Sayaghi (2020), KSA	To determine the compliance of critical care nurses with the ventilator‐associated pneumonia prevention guidelines and the factors that affect their compliance and explore the barriers to implementation of guidelines	Quantitative, cross‐sectional, descriptive study A self‐administered questionnaire of 17 recommended strategies to prevent VAP and 15 barriers Outcomes: Knowledge of VAP CPGs and their use (compliance); barriers faced by nurses in implementing VAP CPGs	8 adult ICUs across 5 hospitals Critical care nurses (*n* = 229) 80.9% response rate	**Barriers:** Staff shortages, forgetfulness, cost control policies in the hospital; compliance was influenced by prior education in ventilator‐associated pneumonia prevention and the capacity (i.e., number of beds) of the intensive care unit **Facilitators:** Not reported
Badparva et al. (2023), Iran	To assess the knowledge of intensive care unit (ICU) nurses on the prevention of Central line‐associated bloodstream infections (CLABSI) and the implementation barriers of evidence‐ based guidelines in practice	Quantitative, cross‐sectional study Self‐administered quantitative survey on knowledge of CLABSI and barriers to guideline use Outcomes: knowledge of ICU nurses on the prevention of CLABSI and barriers to the implementation of CLABSI CPGs	ICUs covering paediatric, adult and neonatal units across 7 hospitals 220 ICU nurses 95% response rate	The median knowledge score of ICU nurses towards CLABSI prevention was 3.00 out of 10, based on a knowledge scale from 0 to 10, with 50.72% scoring < 4 **Barriers:** Median scores are reported, with lower scores reflecting barriers: knowledge deficiency (3.00 out of 10), high workload (4.1 out of 10), shortage of necessary equipment (4.02 out of 10), lack of CLABSI prevention workshops (3.63 out of 10), nursing staff shortage (3.41 out of 10), lack of familiarity with CVC care instructions (3.1 out of 10), lack of attention of nursing managers (2.73 out of 10). **Facilitators:** Not reported
Bankanie et al. (2021), Tanzania	To assess the ICU nurses' knowledge, compliance and barriers towards evidence‐ based guidelines for the prevention of VAP in Tanzania	Quantitative, cross‐sectional study Quantitative, structured, self‐administered questionnaire adopted from a previous study Outcomes: Knowledge, compliance and barriers to CPG use for the prevention of VAP	10 ICUs Intensive care unit nurses (*n* = 116) Response rate not reported	Mean knowledge score was 38.6%; degree status and training level of nurses predicted knowledge **Barriers:** Results presented as percentage of respondents reporting item as a barrier: Lack of skills (96.6%), inadequate staffing (95.5%) and lack of knowledge (79.3%) **Facilitators:** The following were associated with increased knowledge scores or guideline adherence: Higher training level; greater knowledge of guidelines; adequate staffing
Brolliar et al. (2016), USA	To identify and explore in‐depth the provider's perspective on factors associated with adherence to guidelines on paediatric traumatic brain injury (TBI)	Qualitative, exploratory, focus group study Inductive and deductive content analysis of data Outcomes: Factors associated with adherence to guidelines on paediatric TBI	Intensive care unit and emergency department. Nurses and physicians involved in paediatric brain traumatic injury management (*n* = 19 focus groups). (nine focus groups with physicians and 10 focus groups with nurses; a total of 54 physicians and 74 nurses participated)	**Barriers:** Perceived credibility and applicability of guidelines to the individual patients; implementation and dissemination strategies; guideline enforcement; culture, communication and attitudes among providers **Facilitators:** Training and experience in CPG use; local guidelines were preferred
Boan Pion et al. (2021), Switzerland	To assess perinatal palliative care services, examine healthcare professionals' awareness and availability of perinatal palliative care (PnPC) guidelines, and describe HCPs satisfaction with guidelines	Quantitative, nationwide survey Self‐administered quantitative survey including sociodemographic data, perinatal service data, PnPC guideline availability, awareness and satisfaction Outcomes: Facilitators and barriers to guideline‐based care for PnPC; awareness, availability and satisfaction with CPGs	All Swiss tertiary neonatal intensive care units (NICUs) 65% physicians; 49% nurses; 72% psychosocial staff (nurse data was reported separately) Overall response rate 54%	**Barriers:** Insufficient resources for specialised palliative care skills and aftercare in stressful situations were mentioned as significant barriers Training for nurses had limited resources One‐third of nurses were not aware of the existence of PnPC guidelines in their centres Guidelines were often described as ‘too short’, ‘outdated’, or ‘not applied in practice’ Lack of structural feedback mechanisms to improve the practical application of guidelines Guidelines not focusing enough on families and children, with palliative care often seen as termination of all measures **Facilitators:** Training for nurses; feedback within the team; resource allocation to support implementation
Jahansefat et al. (2016), Iran	To evaluate knowledge, attitude and adherence of HCWs to EBGs for prevention of VAP and exploration of the barriers of their implementation in clinical practice	Cross‐sectional study Self‐administered questionnaire including 36 items and open‐ended questions Outcomes: Knowledge and adherence to VAP CPGs and barriers to CPG implementation	Two paediatric cardiac intensive care units Critical care nurses (*n* = 30, 66.7%), paediatricians (*n* = 5, 11.1%), anaesthesiologists (*n* = 4, 8.9%), nurse assistants (*n* = 4, 8.9%) and cardiac surgeons (*n* = 2, 4.4%) Response rate 84.9%	**Barriers:** 1. Individual factors: Negative personality traits, negative attitude, lack of moral responsibility, low levels of knowledge, inappropriate personal values irreligiousness 2. Organisational factors: Lack of time, lack of facilities, lack of sufficient support, weak leadership, lack of supervision, cost‐effectiveness issues, lack of collaboration, ethical issues 3. Educational factors: Complication in recommendations, ineffective academic education, curriculum issues, post‐graduation education limitations 4. Social factors: Economic problems, lack of governmental support **Facilitators:** Not reported
Janssen et al. (2012), the Netherlands	To identify factors that influence the implementation of the guideline Triage in emergency departments [2004] in emergency departments in the Netherlands, and, to develop tailored implementation strategies for implementation of this guideline	Cross‐sectional, descriptive design Qualitative findings of an initial mixed methods study: focus groups and in‐depth interviews (qualitative) Questionnaires on knowledge and self‐reported adherence to triage CPGs	Questionnaires completed by staff (no breakdown of staff type) (*n* = 108) with a 75% response rate + Qualitative focus groups comprising emergency department nurses, and ward managers (*n* = 25, of whom 18 were nurses)	**Barriers:** Lack of resources, including lack of triage room, education and personnel and high workload; nurses were reluctant to perform triage where conditions were inadequate; in focus groups, nurses noted barriers as the social context, including colleague resistance and difficulty in overcoming resistance and commitment to perform triage without support from physicians. Other influencing factors included lack of feedback, lack of interprofessional support, lack of knowledge and skills and lack of a clear protocol **Facilitators:** Adequate resources: training to enhance knowledge and skills; protocols for guideline use
Jansson et al. (2013), Finland	To explore critical care nurses' knowledge of adherence to and barriers to evidence‐based guidelines for the prevention of VAP	Quantitative, cross‐sectional study Two multiple‐choice questionnaires to assess critical care nurses' knowledge, attitudes and barriers to VAP CPGs Outcomes: Knowledge, attitudes and barriers to VAP CPGs	Single academic centre in Finland Critical care nurses (*n* = 101) Response rate 56.4%	**Barriers:** Disagreement with results of guideline‐based care (16.9%), time availability (14.3%), lack of skills (11.9%), knowledge (7.1%) and guidance (7.1%) **Facilitators:** Nurse experience was linked to better performance on knowledge of guidelines (*p* = 0.029)
Katz et al. (2014), USA	To characterise emergency physicians' and nurses' perceptions of smoking cessation counselling and to identify barriers and facilitators to implementation of the 5 A's framework (Ask‐Advise‐Assess‐Assist‐Arrange) in the ED	Qualitative Semi‐structured interviews, face‐to‐face, data analysed using content analysis Outcomes: Perceptions of cessation counselling and barriers and facilitators to smoking cessation guideline implementation	A single university hospital Emergency department physicians (*n* = 11) and nurses (*n* = 19) Response rate not reported	**Barriers:** Time constraints, competing care demands and patient resistance influenced smoking cessation guideline perceptions for nurses **Facilitators:** Training; collaborative practice; improved workflow
Kiyoshi‐Teo et al. (2014), USA	To identify factors that influence adherence to guidelines for the prevention of VAP, with a focus on oral hygiene, head‐of‐bed elevation and spontaneous breathing trials	Quantitative, cross‐sectional study Self‐administered quantitative survey including examination of facilitators and barriers (user, guideline or contextual) for CPGs related to VAP Outcomes: Facilitators and barriers to guideline‐based care of VAP, with a focus on oral hygiene, head‐of‐bed elevation and trials of spontaneous breathing	Critical care nurses across 8 hospitals in California (18 ICUs) Critical care nurses (*n* = 576) Response rate 44.6%	**Barriers:** Odds ratios for adherence to VAP prevention guidelines: Guideline awareness (1.30); lack of time (1.54); poor quality guidelines (1.20); prioritisation of use (1.07) **Facilitators:** Positive attitudes towards guidelines were the strongest predictor of use (odds ratio 3.49–4.75)
Mpasa et al. (2023), Malawi	To evaluate Malawian critical care nurses' views on the implementation of an endotracheal tube cuff pressure management guideline to enhance sustained guideline use	Questionnaire including open‐ended and closed questions Outcomes: Facilitators and barriers to guideline‐based care and wider views on CPGs related to endotracheal tube cuff pressure management	6 ICUs in 6 hospitals in Malawi Critical care nurses (*n* = 47) Response rate 77%	**Barriers:** Resource constraints—Participants identified the need for specific resources, such as manometers for monitoring endotracheal cuff pressure Incomplete follow‐up and supervision—Participants recommended continuous supervision and follow‐up post‐implementation of guidelines, suggesting a perceived lack of ongoing support and oversight **Facilitators:** Positive evaluation of implementation strategies—Participants generally found the implemented strategies (half‐day educational sessions, printed materials and monitoring visits) useful, clear and applicable. This positive perception among participants can facilitate guideline implementation and uptake Recommendations for education and training—Nurses emphasised the importance of education and training for ICU staff on practical guideline application
Noome et al. (2017), the Netherlands	To examine the effectiveness of supporting intensive care units on implementing guidelines on end‐of‐life care	Quantitative, cluster randomised controlled trial Self‐administered quantitative survey for nurses, interviews with nurses and questionnaire for family members of deceased patients Outcomes: Adherence to guidelines based on the use of a supportive programme for implementation; satisfaction; unmet needs and barriers	8 ICUs (intervention) and 5 ICUs (control) Intensive care nurses (*n* = 231) Response rate 32%	**Barriers:** Negative departmental culture influenced guideline implementation and adherence; low belief in guideline use and value; negative leadership and interprofessional team dynamics informed culture **Facilitators:** Effective leaders, tailored education and reminders, audits and feedback were facilitators of guideline uptake; simplified formats, prioritisation of guidelines and electronic media may support guideline implementation
Ricart et al. (2003), Spain	To review barriers to nursing adherence to nonpharmacologic evidence‐based guidelines for preventing VAP	Quantitative, descriptive study questionnaire Self‐administered quantitative survey assessing adherence to 19 non‐pharmacological strategies within guidelines Outcomes: Barriers to adherence of CPGs for non‐pharmacological prevention of VAP	Intensive care units 110 nurses approached at two critical care nurse meetings Response rate 46.3%	**Barriers:** Unavailability of resources accounted for 37% of barriers, others included patient discomfort (8.2%) and disagreement with results of the guidelines (7.8%); patient‐related barriers, guideline‐related barriers, overwork or lack of time and environmental barriers were also noted by a small proportion of respondents **Facilitators:** Not reported
Richards et al. (2024), Australia	To examine the perspectives of ED staff who respond to self‐harm presentations, perceived barriers to providing optimal, guideline‐concordant care and staff's familiarity with existing guidelines	Quantitative, cross‐sectional study Quantitative survey based on the attitudes to self‐harm questionnaire Outcomes: Familiarity with guidelines, barriers to guideline use and concordance with guidelines on self‐harm presentations	Emergency departments from 2 hospitals The survey was completed by 131 staff (83.2% nurses) Response rate 16 (hospital 1) 37% (hospital 2)	**Barriers:** Staff noted lack of space (62.3%) and time (78.7%) to conduct the appropriate assessments, lack of self‐harm training (71.8%) and limited awareness of or access to guidelines and recommendations for self‐harm management within the ED (63.6%) **Facilitators:** Not reported
Schechter‐Perkins et al. (2014), USA	To assess staff knowledge of Centres for Disease Control (CDC) guidelines, attitudes and barriers towards emergency department (ED) HIV testing before and after implementing an ED rapid HIV testing programme (EDRHTP)	Quantitative, cross‐sectional pre/post survey Self‐administered quantitative survey assessing familiarity, knowledge and barriers to guideline use following EDRHTP Outcomes: Knowledge, attitudes, facilitators and barriers to guideline‐based care related to implementation of an EDRHTP	Emergency department registered nurses (RNs) and doctors (MDs) at a single centre The pre‐programme survey was completed by 124/132 (94%) staff, which included 60 MDs and 64 RNs The post‐programme survey was completed by 148/160 (93%) staff, including 77 MDs and 71 RNs	Pre‐Programme Implementation: **Barriers:** 56% identified at least one potential barrier towards an EDRHTP: Cost concerns (15%) Confidentiality concerns (56%) Lack of follow‐up (22%) Interference with patient flow (52%) Post‐Programme Implementation: Awareness and Perception: 95% of staff were aware of the current testing programme Increased awareness of the 2006 CDC guidelines (59% vs. 44%, *p* = 0.04) Continued perception that HIV is a major problem (91% vs. 93%, *p* = 0.8) **Facilitators:** Implementation of a structured education/training programme; staff and patient support for interventions within guidelines 133 (94%) respondents supported HIV testing by separate counsellors
Sinuff et al. (2007), Canada	To understand clinicians' perceptions regarding practice guidelines in Canadian ICUs to inform guideline development and implementation strategies	Quantitative, cross‐sectional study Self‐administered, quantitative survey Outcomes: Views on local ICU guideline development and use, attitudes and preferences towards guidelines	National survey across multiple ICUs 565 respondents in total (*n* = 442 nurses; data reported separately) Response rate 51.6%	**Barriers:** Lack of awareness about the existence of guidelines, lack of familiarity with guideline content, lack of familiarity with the benefit associated with interventions described in the guideline, lack of motivation to change, perception of risk associated with guideline implementation, lack of access to guidelines, lack of formal implementation strategies at the institution, lack of institutional prioritisation for guidelines, lack of time **Facilitators:** Not reported
Trogrlic et al. (2017), the Netherlands	To identified barriers for implementation that should be addressed in a tailored implementation intervention targeted to improved ICU delirium guideline adherence	Quantitative, cross‐sectional study Self‐administered quantitative survey aimed at identifying barriers for implementation for guidelines in the ICU Outcomes: Barriers to delirium guideline‐based care in the ICU	Intensive care units in 6 hospitals in the Netherlands Physicians 53 (15%), ICU nurses 283 (79%) Response rate 64%	**Barriers:** Knowledge gaps: Mean delirium knowledge score was 64% for all respondents, with nurses scoring lower (61%) compared to physicians (72%) and delirium experts (75%) Limited training and education: Only 37% of respondents had received bedside teaching about delirium, and 39% had participated in ICU delirium‐related training in the past 3 years Collaboration challenges: < 50% of physicians and nurses felt that nurses were satisfied with physician‐initiated delirium management. Moreover, only 47% felt confident about what to do when the CAM‐ICU was positive Guideline adherence issues: Only 21% of respondents were familiar with the Dutch ICU delirium guideline. Barriers to adherence included the perception that guidelines did not result in optimal care, unwillingness to change practices, lack of time and seeing the guidelines as cumbersome Absence of a workable protocol for the delirium guideline and previous implementation efforts being insufficient as barriers **Facilitators:** Not reported
Yeganeh et al. (2019), Iran	To assess intensive care unit nurses' knowledge of evidence‐based guidelines for VAP prevention	Quantitative, cross‐sectional study Quantitative survey comprising three parts: demographic data, knowledge of guidelines for VAP and barriers to guideline implementation Outcomes: Knowledge of VAP guidelines and barriers to their implementation in ICU nurses	14 ICUs across 11 hospitals 171 nurses Response rate 78.1%	**Barriers:** Lack of equipment (Endotracheal tubes with subglottic suctioning paths and kinetic beds) Limited educational resources: Compiled education and educational seminars about evidence‐based guidelines for the prevention of VAP were available only in five units Knowledge gaps: The mean knowledge score for the nine items was 4.63 ± 1.708, indicating that only 51.4% of the answers were correct Work experience: Although nurses with more than 10 years of experience showed higher levels of knowledge, the difference was not statistically significant (*p* = 0.327). This suggests that experience alone may not be Insufficient training: Only 41.1% of the nurses had taken intensive care training courses. The lack of mandatory or widespread training courses can hinder the standardisation and effectiveness of VAP prevention practices Workload: A significant portion of nurses (43.9%) were working in units with 6 to 12 beds. Larger unit sizes with potentially higher patient‐to‐nurse ratios can impede the consistent application of evidence‐based VAP prevention guidelines due to workload and time constraints **Facilitators:** Not reported

**TABLE 3:** Summary of the quality assessment outcomes.

	Score	Rating
Qualitative studies		
Brolliar et al. (2016)	7/10	Moderate
Janssen et al. (2012)	8/10	High
Katz et al. (2014)	6/10	Moderate
Cross‐sectional studies		
Al‐Sayaghi (2020)	7/8	High
Badparva et al. (2023)	8/8	High
Bankanie et al. (2021)	7/8	High
Boan Pion et al. (2021)	8/8	High
Jahansefat et al. (2016)	7/8	High
Jansson et al. (2013)	8/8	High
Kiyoshi‐Teo et al. (2014)	8/8	High
Mpasa et al. (2023)	8/8	High
Ricart et al. (2003)	6/8	High
Richards et al. (2024)	7/8	High
Schechter‐Perkins et al. (2014)	8/8	High
Sinuff et al. (2007)	8/8	High
Trogrlic et al. (2017)	8/8	High
Yeganeh et al. (2019)	7/8	High
Randomised Controlled Trials		
Noome et al. (2017)	6/13	Moderate

Exploration of the barriers and facilitators to the implementation of CPGs among nurses in the ED and CCU settings was captured in three themes derived from the data set. These themes were identified as: (1) individual factors. They included awareness, education, training, knowledge and skills of the involved medical practitioners. In turn, (2) CPG‐related factors included the content, design and characteristics of the guidelines. Finally, (3) organisational factors were presented by leadership support, availability of resources, time management, workload, the levels of staffing and role clarity (Table 4). It should be noted, however, that the majority of studies are focused on the identification of barriers to CPG implementation, with only a few studies addressing facilitators.

**TABLE 4:** A summary of the identified themes from across the literature.

Theme	Factors
Individual factors	Awareness of guidelines Beliefs aligning with guideline content Resistance to change or innovation Level of knowledge and skills Motivational level Perceived colleague support and enthusiasm
Clinical practice guideline factors	Complexity of format or pathway Relevance to patients seen in practice Degree to which guidelines were user‐friendly Length of guidelines and documentation Time to complete guidelines
Organisational factors	Poor role clarity or definition Lack of training/inadequate training Implementation strategy and change management Department culture Multidisciplinary rounds and team dynamics Resource availability Workload and staffing levels

### 4.1 Individual Factors

Individual factors emerged as a key source of barriers and facilitators to CPG implementation, comprising factors that acted at the level of the individual healthcare professionals, including lack of knowledge, lack of awareness, attitudes, work preferences, training, education, knowledge and skills development.

A common barrier to CPG implementation in the ED was lack of knowledge and awareness of CPGs (Janssen et al. 2012; Richards et al. 2024). Two‐thirds of nurses expressed limited awareness of and access to guidelines in one study (Richards et al. 2024), with another study reporting awareness of guidelines at only 44% among nurses (Schechter‐Perkins et al. 2014). Lack of awareness was consistently noted across studies, regardless of the topic of CPGs. Additionally, awareness of CPGs was low in studies focusing on nursing staff in the CCU setting (Kiyoshi‐Teo et al. 2014; Trogrlic et al. 2017; Boan Pion et al. 2021; Badparva et al. 2023). One study found that only 30% of nurses achieved scores on assessment consistent with an understanding of CPG‐based care (Badparva et al. 2023). Other studies confirmed this observation (Yeganeh et al. 2019; Jansson et al. 2013; Bankanie et al. 2021), with a reported 51.4% of questions answered correctly in one analysis of ventilator‐associated guidelines (Yeganeh et al. 2019).

Attitudes and work preferences also influenced CPG implementation among nurses. Negative attitudes may arise towards CPGs where they have poor perceived credibility, value, or relevance to the specific practice setting (Sinuff et al. 2007; Brolliar et al. 2016; Jahansefat et al. 2016). This is particularly problematic when CPGs were considered outdated, or where they deviated from norms or routine practice, reflecting staff reluctance to engage in practice change or innovation (Kiyoshi‐Teo et al. 2014; Noome et al. 2017; Boan Pion et al. 2021). Attitudes favouring individualised decision‐making may reduce reliance or perceived value of CPGs, depending on their breadth and depth (Janssen et al. 2012; Brolliar et al. 2016). Attitudes of colleagues may also influence nurses regarding the motivation and intention to use CPGs; when nursing colleagues were not supportive or held negative attitudes towards CPGs, this reduced motivation to engage with them and implement their recommendations (Sinuff et al. 2007; Noome et al. 2017).

Facilitators of CPG use at the individual level included high‐quality education and training, which could target deficits in skills and knowledge and was associated with improvement in CPG awareness and knowledge, as well as more positive attitudes towards CPGs (Brolliar et al. 2016; Mpasa et al. 2023). Support from colleagues and other staff was also associated with more positive attitudes towards CPGs (Noome et al. 2017).

Complementing the above, effective facilitators for the implementation of CPG at the individual level included structured education and training, which may further enhance confidence in guidelines, knowledge, awareness and adherence to the guidelines. Supportive peer environments and leadership interventions also encourage an overall positive attitude towards CPGs. Finally, mentorship programmes, interactive learning and hands‐on workshops can further strengthen the level of engagement with the patients, leading to reinforced evidence‐based practice in the analysed clinical settings (Brolliar et al. 2016; Mpasa et al. 2023).

### 4.2 Clinical Practice Guideline Factors

This theme captured the aspects of the design of CPGs that may have an influence in implementation. Identified barriers included the complexity, length, time needed to implement, relevance and user‐friendliness of the guideline, while facilitators included simplified CPG formats and supportive resource use.

One barrier to CPG use was the content of CPGs, where content that did not apply to the local setting or contrasted with local recommendations led to a risk of CPGs being deemed irrelevant and then not implemented (Katz et al. 2014; Brolliar et al. 2016; Trogrlic et al. 2017). Similarly, when CPGs failed to define roles and responsibilities of nursing staff within a care pathway, this was associated with lower perceived relevance and value/credibility of CPGs (Janssen et al. 2012; Katz et al. 2014). This was also true of CPGs with outdated content or those that failed to exhibit content relevant to contemporary trends or practices (Boan Pion et al. 2021).

The design of CPGs was also a notable barrier to their implementation (Sinuff et al. 2007; Trogrlic et al. 2017) especially if they were viewed as cumbersome or difficult to implement in a short period of time. The complexity of the format and recommendations presented in CPGs was a widely noted barrier to their use (Sinuff et al. 2007; Jahansefat et al. 2016; Boan Pion et al. 2021). Overly complex, CPGs may interfere with the flow of patient care, with 44% of nurses in one study observing that flow was interrupted and delays in care were introduced in the ED with CPG use (Schechter‐Perkins et al. 2014). This may reflect initial challenges in introducing CPGs and the need for staff to adapt to their use, rather than inefficiencies embedded within CPGs; however.

Enhancing the user‐friendliness of CPGs was linked to the facilitation of their implementation, including reducing the number of documents to complete, abbreviation or streamlining of documents, which reduced the time needed to review and use these documents (Brolliar et al. 2016; Mpasa et al. 2023). Additionally, the use of supportive resources for CPGs was also advantageous in promoting their use, including electronic media and algorithms to support rapid decision‐making (Sinuff et al. 2007). The relevance of CPGs to the local care setting, including localised recommendations, was also considered favourably by nurses (Brolliar et al. 2016).

Expanding the above, primary facilitators for CPG include the availability of concise, clear and visually structured guidelines that both enhance usability and minimise complexity. At the same time, the integration of digital tools, such as mobile‐accessible protocols and software focused on decision support, improves the level of adherence. The implementation of localised adaptations and interactive training, along with regular feedback, further supports nurses in applying CPGs effectively, ensuring both relevance and effective practice (Brolliar et al. 2016).

### 4.3 Organisational Factors

Numerous organisational factors may serve as barriers or facilitators to CPG implementation, including role clarity, workload, resources, change management and leadership. Role clarity was considered an important factor in clarifying the responsibilities of nurses within CPGs; where these responsibilities were unclear, CPG adherence and engagement were low (Janssen et al. 2012) and were considered a barrier to their use (Sinuff et al. 2007; Brolliar et al. 2016; Noome et al. 2017; Yeganeh et al. 2019).

Workload, staffing levels and resources were also considered barriers to CPG implementation. Workload, low staff numbers and limited resources may lead to time constraints for care, reducing adherence to guidelines, which was observed in both ED and CCU settings (Ricart et al. 2003; Katz et al. 2014; Trogrlic et al. 2017; Al‐Sayaghi 2020; Badparva et al. 2023). In one study, 35%–40% of nurses expressed staff shortages and high workloads as problematic (Badparva et al. 2023). Limited staffing was also linked to poor supervision of colleagues and lack of collaborative practice embedded within CPGs (Jahansefat et al. 2016).

Resource limitations and needs were also evident, including protocols and equipment for care delivery and education and training resources (Al‐Sayaghi 2020; Badparva et al. 2023). In one study (Richards et al. 2024) resource constraints included lack of space (62.3%) and lack of time (78.7%), which were linked to staffing levels and workloads. Lack of triage rooms and care resources was noted (Janssen et al. 2012) while 37% of nurses reported that resource scarcity limited adherence to guidelines on ventilator‐associated pneumonia (Ricart et al. 2003). In one study of low‐income settings, the quality of resources and their cost‐effectiveness were also considered a barrier to CPG implementation (Mpasa et al. 2023).

Other important factors included the process of introducing and supporting CPGs from a leadership perspective, where lack of support and inappropriate change culture impeded implementation (Janssen et al. 2012; Brolliar et al. 2016). Departmental culture and leadership were noted as barriers in several studies (Sinuff et al. 2007; Noome et al. 2017). Furthermore, a lack of enforcement of guidelines by staff and leaders was evident (Brolliar et al. 2016).

In the context of the above, leadership support at multiple levels, such as hospital administration, unit managers and individual nursing staff, is essential for efficient CPG implementation. Specifically, strong leadership plays a fundamentally important role in clinical settings as it is able to facilitate the allocation of resources, reinforce guidelines and change management in cases when a more effective one is necessary (Brolliar et al. 2016).

These key facilitators of CPG implementation included effective management and leadership (Sinuff et al. 2007), colleague support and motivation (Noome et al. 2017) and effective training and education. The role of training and education was particularly important across studies, with studies reporting that only 28%–50% of those surveyed felt training was adequate (Yeganeh et al. 2019; Richards et al. 2024). Guideline uptake and adherence were enhanced with training and education strategies; had positive effects on implementation (Sinuff et al. 2007; Al‐Sayaghi 2020; Badparva et al. 2023).

## 5 Discussion

This review identified the known barriers and facilitators to implementation of CPGs among nurses in ED and CCU settings. The results showed that nurses perceive individual, CPG‐related and organisational factors acted as both barriers and facilitators to CPG implementation. Some of the key barriers noted at the individual level were a lack of awareness and knowledge among nursing staff, lack of skills, negative attitudes and lack of support or motivation. Furthermore, barriers related to CPGs included the content and design of guidelines, which may be cumbersome or time‐consuming. Finally, barriers linked to organisational factors were numerous but generally reflected a lack of education and training, leadership and support and resources for CPG implementation. In contrast, facilitators across these themes included colleague support, staff training and education, streamlined or user‐friendly CPG design and support from leadership.

The conducted review highlights the fact that while facilitators such as leadership encouragement, training and colleague support exist, barriers to the implementation of CPG were more frequent across organisational, CPG‐related and individual domains. Consequently, the predominance of barriers points out the need for targeted interventions that would enhance facilitators and improve CPG adherence in the corresponding nursing practice.

These findings are broadly consistent with previous literature on the topic, where numerous barriers have been noted to influence CPG uptake; although key differences exist in healthcare settings, assessed populations and study contexts (Ebben et al. 2013; Holt and Vardaman 2013; Rafferty et al. 2013). However, the specific focus on nurses in the ED and CCU settings emphasises the importance of this review and the potential implications for CPG use in this context. Awareness and knowledge of CPGs have been associated with acceptance of guidelines and their use, including guidelines related to paediatric traumatic brain injury (Brolliar et al. 2016), ventilator‐associated pneumonia (Al‐Sayaghi 2020) and perinatal palliative care (Boan Pion et al. 2021). These findings were not only seen across various conditions but also consistent across nations, such as the United States (Brolliar et al. 2016), Finland (Jansson et al. 2013) and Tanzania (Bankanie et al. 2021). Awareness and knowledge are closely related to organisational factors, such as a lack of training or support for guidelines, which may impact on awareness and knowledge (Jansson et al. 2013; Brolliar et al. 2016). Therefore, targeting enhanced training and support for staff may be important in overcoming deficits in awareness and knowledge, as demonstrated in wider literature. For example, the respective literature supports the findings of the review, highlighting that both education and training are critical for improving CPG adherence (Tudor Car et al. 2019; White et al. 2019).

Targeting knowledge and awareness alone may have limitations in promoting CPG implementation, as negative attitudes and lack of support in terms of staffing and resources may also influence implementation, despite awareness of these CPGs. Based on the above, the wider literature emphasises that knowledge alone is insufficient for CPG adherence because a combination of workplace culture, behavioural intentions, and attitudes is also known to play an important role (Armitage and Conner 2001; Keyworth et al. 2020; MacLennan et al. 2022). Addressing attitudes and improving support may justify an approach that targets the organisational culture of the care setting, with leadership, management and frontline staff impacted by the change. Nevertheless, the corresponding findings are based on broader healthcare settings, whereas the review emphasises that nurses in CCU and ED environments require structured leadership support (Cook et al. 2018). Engagement at every level of the healthcare system, from frontline nursing staff to leadership, plays a highly important role in fostering a supportive approach where the implementation of CPGs is not only encouraged but is also considered sustainable, ensuring awareness, training and correct allocation of resources in the practical environment of both ED and CCU (Hayes 2018; White et al. 2019). However, engaging frontline nursing staff in changes and in strategies to support CPG use will also be important to ensure that their needs are met (Hayes 2018). Furthermore, wider organisational approaches to managing workload, ensuring adequate staffing levels and providing support and resources for CPG use will also be needed at a practical level (Niessen et al. 2018; Romanowski et al. 2020). However, in contrast to the review, both of these studies are focused on the general healthcare environments with no discussion of ED/CCU challenges such as reliance on leadership support and time‐sensitive patient care. The specific needs of staff will vary according to the context of the care environment, local resource needs and the topic of the CPG, necessitating an adaptive approach.

Finally, changes to the content and design of CPGs are an important consideration in not only ensuring they are accurate and up‐to‐date but also promoting perceptions of utility and value for their use, as well as integrating them more successfully into established workflows. Nurses with negative attitudes towards CPGs often expressed frustration with a lack of focus on local challenges, the role of the nurse, poor design, outdated recommendations, or failure of recommendations to align with established practice (Moore et al. 2017; Yeganeh et al. 2019; Wager et al. 2021). Therefore, CPGs should be developed with these factors in mind and, where possible, local modifications and adaptations should be considered to maximise relevance and value to specific ED and CCU settings (Miao et al. 2015; Craig et al. 2016; Batchelor et al. 2023). When introduced to the clinical area, a CPG also needs to be accessible and easy to use, with the use of electronic media (algorithms, decision support tools) and streamlined guideline content advocated in this review (Kuhn et al. 2015; Meijer et al. 2019). Therefore, CPG implementation cannot be assumed to reflect a lack of willingness or motivation to adopt new guidelines but should be critical of CPG content and design as well.

The findings above suggest that effective CPG implementation is supported by a combination of structured education, leadership engagement and streamlined guided design. At the same time, the adoption can be increased through the enhanced usage of workplace support systems, peer mentorship and digital tools. Finally, it is expected that the level of acceptance can be increased by addressing gaps in staff training and accessibility of resources. The facilitators addressed ensure that CPGs become integral components of daily practices in clinical settings.

Finally, the review determined no considerable differences in the barriers or facilitators to the implementation of CPG between the ED and CCU settings. Nevertheless, there was relatively less literature obtained/analysed on the ED settings, pointing out a possible research gap. Further investigations are necessary to establish whether settings‐specific challenges have the potential to influence CPG adherence in emergency care environments.

## 6 Strengths and Limitations

The validity of this review was considered throughout the phases of identifying, analysing and synthesising the literature. The use of a systematic process across these areas increased the reliability and rigour of the findings compared with other review techniques (Parahoo 2014). The use of two reviewers to independently identify studies and perform quality assessment added to validity by minimising bias (Ma et al. 2020). The focus of the review was specifically on nursing staff in ED and CCU settings, which allowed for a refined search and analysis that maintained validity to the staff population and care context.

One limitation of the review is the inclusion of English language studies only, which may have excluded data from across the globe. This decision was justified to reduce translation costs and reduce error risk (Nussbaumer‐Streit et al. 2020); however, it may have led to bias in the findings. In addition, data extraction was performed by a single reviewer, which may have reduced the reliability of this stage. However, each step of the review was discussed with the review team, and adherence to PRISMA guidance was intended to minimise this bias (Ma et al. 2020).

The findings of the review comprised 18 studies with notable heterogeneity of methods, populations and the type of CPGs assessed; these factors may have influenced the comparability of data and the validity of the synthesis approach (Aveyard et al. 2021). In addition, the barriers and facilitators identified may be context‐dependent, reflecting local challenges and specific nursing needs or attitudes, which may limit wider generalisability. For instance, data from low‐income nations (Mpasa et al. 2023) may not be relevant in high‐income settings, where resources and training needs may differ. Finally, there was a notably small number of facilitators identified compared with barriers, which may reflect a lack of data saturation and could influence the generation of valid recommendations for improving CPG implementation (Aveyard et al. 2021).

## 7 Conclusion

This review provides a comprehensive and contemporary analysis of literature focused on barriers and facilitators to the implementation of CPGs among nurses in ED and CCU settings. A total of 18 studies were identified and led to the identification of three themes, reflecting individual, CPG‐related and organisational barriers and facilitators to CPG use. The findings highlight the need to target awareness, knowledge, attitudes and resource or support needs through targeted training and education, as well as wider management/leadership approaches and engaging approaches to CPG design and content development. The interplay of individual, CPG‐related and organisational factors was notable in the published literature, suggesting that no single approach can be easily targeted without consideration of the others. Hence, implementation of CPGs needs to reflect the holistic approaches of change management, staff motivation, training and education and wider support for CPG use in the local context.

## 8 Relevance to Clinical Practice

From a practice perspective, this review highlights the importance of considering specific barriers to CPG implementation for nursing staff in ED and CCU settings. Unique factors may have an impact on CPG use in these contexts and should be targeted in a focused manner. Equally important, the review highlights the facilitators of CPG use, which may be introduced more widely in practice, including the need for CPG development and design that can be introduced in time‐sensitive care contexts, training and education on CPGs focused on raising awareness and improving knowledge and skills, and wider organisational approaches to providing support and promoting a positive culture for CPG acceptance and implementation.

## Conflicts of Interest

The authors declare no conflicts of interest.

## Supporting information


**Data S1:** jocn70086‐sup‐0001‐Supinfo01.docx.

## Data Availability

The authors have nothing to report.
